# Genetic basis of photosynthetic responses to cold in two locally adapted populations of *Arabidopsis thaliana*

**DOI:** 10.1093/jxb/erx437

**Published:** 2017-12-28

**Authors:** Christopher G Oakley, Linda Savage, Samuel Lotz, G Rudd Larson, Michael F Thomashow, David M Kramer, Douglas W Schemske

**Affiliations:** 1 Department of Plant Biology, Michigan State University, East Lansing, MI, USA; 2 MSU-DOE Plant Research Laboratory, Michigan State University, East Lansing, MI, USA; 3 Department of Biochemistry and Molecular Biology, Michigan State University, East Lansing, MI, USA; 4 Genetics Graduate Program, Michigan State University, East Lansing, MI, USA; 5 Plant Resilience Institute, Michigan State University, East Lansing, MI, USA; 6 W.K. Kellogg Biological Station, Michigan State University, East Lansing, MI, USA

**Keywords:** Adaptation, chlorophyll fluorescence, cold acclimation, *F*_v_/*F*_m_, genotype by environment interaction, natural variation, non-photochemical quenching, photoprotection, photosynthesis, QTL mapping

## Abstract

Local adaptation is common, but the traits and genes involved are often unknown. Physiological responses to cold probably contribute to local adaptation in wide-ranging species, but the genetic basis underlying natural variation in these traits has rarely been studied. Using a recombinant inbred (495 lines) mapping population from locally adapted populations of *Arabidopsis thaliana* from Sweden and Italy, we grew plants at low temperature and mapped quantitative trait loci (QTLs) for traits related to photosynthesis: maximal quantum efficiency (*F*_v_/*F*_m_), rapidly reversible photoprotection (NPQ_fast_), and photoinhibition of PSII (NPQ_slow_) using high-throughput, whole-plant measures of chlorophyll fluorescence. In response to cold, the Swedish line had greater values for all traits, and for every trait, large effect QTLs contributed to parental differences. We found one major QTL affecting all traits, as well as unique major QTLs for each trait. Six trait QTLs overlapped with previously published locally adaptive QTLs based on fitness measured in the native environments over 3 years. Our results demonstrate that photosynthetic responses to cold can vary dramatically within a species, and may predominantly be caused by a few QTLs of large effect. Some photosynthesis traits and QTLs probably contribute to local adaptation in this system.

## Teaser

Dramatic intraspecific natural variation in photosynthetic responses to cold is controlled mainly by a few large effect QTLs, and some cold-responsive photosynthesis QTLs may contribute to local adaptation.

## Introduction

In broadly distributed plant species, populations are typically exposed to divergent selection due to spatial variation in biotic and abiotic factors. Evidence for local adaptation, where native genotypes outperform foreign genotypes, is now extensive (reviewed in Hereford, 2009).

More recently, studies have employed genetic mapping approaches to identify the genetic basis of local adaptation (Lowry *et al.*, 2009; Anderson *et al.*, 2011*a*; Ågren *et al.*, 2013; Leinonen *et al.*, 2013; Postma and Ågren, 2016). These studies may shed light on major questions about the genetics of adaptation, such as the number and effect sizes of mutations underlying adaptation (reviewed in Dittmar *et al.*, 2016), and whether or not locally adaptive alleles result in fitness trade-offs in alternative environments (Anderson *et al.*, 2011*b*). Using these systems to investigate the genetic basis of adaptive traits further provides an opportunity to gain a mechanistic understanding of adaptive differentiation (Lowry *et al.*, 2009; Anderson *et al.*, 2011*a*; Oakley *et al.*, 2014; Ågren *et al.*, 2017: Postma and Ågren, 2016).

A number of studies have mapped the genetic basis of plant morphological and phenological traits (Alonso-Blanco and Méndez-Vigo, 2014). However, few studies have investigated the genetic basis of physiological traits (Flood *et al.*, 2011). This is surprising given the importance of physiological adaptation in plants (McKay *et al.*, 2003; Lowry *et al.*, 2009; Preston and Sandve, 2013) and is probably due to the difficulty in obtaining precise physiological measurements for a large number of individuals, as is required for genetic mapping studies. Our poor understanding of the genetic basis of physiological traits undermines efforts to reach a general consensus on the genetic architecture of adaptation.

Plant physiological adaptations often include traits related to cold acclimation (Thomashow, 2010; Preston and Sandve, 2013). While the ability to withstand freezing is likely to be involved in adaptation to cold environments (Zhen and Ungerer, 2008; Oakley *et al.*, 2014), photosynthetic responses to cold, but non-freezing, temperatures can be important as well. Under conditions of high photosynthetically active radiation (PAR), or under adverse environmental conditions that restrict the rate of photosynthesis, photon capture can exceed the rate at which the energy can be used, resulting in production of reactive oxygen species (ROS) and subsequent functional inactivation, and even cell damage (Demmig-Adams and Adams, 2006; Tikkanen *et al.*, 2012). While light harvesting is a temperature-independent process, utilization of excitation energy in the biochemistry of photosynthesis is depressed at lower temperatures (Demmig-Adams *et al.*, 2012; Hüner *et al.*, 2012). Winter annuals can take advantage of non-freezing days that are conducive to photosynthesis, but need the ability to dissipate excess excitation when light intensity exceeds that which can be utilized at a given temperature. Plants dissipate excess light energy as heat through processes called non-photochemical quenching (NPQ) including: qE, which is regulated by acidification of the thylakoid lumen (Müller *et al.*, 2001; Demmig-Adams and Adams, 2006; Brooks *et al.*, 2014; Strand and Kramer, 2014); qZ, related to the accumulation of zeaxanthin (Nilkens *et al.*, 2010); and qI, the inactivation and/or removal of PSII reaction centers (Aro *et al.*, 1993; Demmig-Adams *et al.*, 2012; Demmig-Adams *et al.*, 2014), as well as state transitions (Mullineaux and Emlyn-Jones, 2005; Hogewoning *et al.*, 2012). The NPQ processes act as excitation ‘release valves’, decreasing excitation pressure and accumulation of strongly reactive intermediates of photosynthesis, but at a potential cost of lost energy upon return to limiting light (Müller *et al.*, 2001; Demmig-Adams *et al.*, 2012).

Plants must therefore regulate photoprotection to balance their often-competing needs for efficient capture of light energy and carbon fixation, and the avoidance of photodamage. The genes that control this balance are almost certainly under selective pressure to optimize photosynthetic responses to local environmental conditions. Indeed, plant species differ greatly in the degree to which they modulate photosynthesis in response to environmental cues (Long *et al.*, 2006; Kramer and Evans, 2011; Hüner *et al.*, 2012). For example, plants from colder regions tend to show enhanced NPQ mechanisms, that decrease excitation pressure under chilling temperatures (Hüner *et al.*, 1993; Demmig-Adams and Adams, 2006; Demmig-Adams *et al.*, 2012). The extent of natural variation among populations of the same species in photosynthetic parameters, either constitutive differences or plastic responses to environmental cues, is understudied (Flood *et al.*, 2011). Such intraspecific variation can be an important tool for studying the genetic basis of photosynthetic traits and their potential contribution to adaptive differentiation.

Here, we present the results of a genetic mapping study designed to identify quantitative trait loci (QTLs) involved in the physiological response to cold temperature in the model plant *Arabidopsis thaliana* (hereafter Arabidopsis). We used a large mapping population of recombinant inbred lines (RILs) produced from a cross between lines from two Arabidopsis populations. One population is from Sweden (Rödåsen; N62°48', E18°12') near the northern edge of the native range where freezing temperatures are common, and one population is from Italy (Castelnuovo; N42°07', E12°29'), near the southern edge of the native range where freezing is rare. Both populations are winter annuals growing on open hillsides, and both experience cold but non-freezing conditions prior to winter.

Reciprocal transplant experiments carried out over 5 years demonstrated that these populations are locally adapted (Ågren and Schemske, 2012). Subsequent QTL mapping of fitness in the field at both native sites identified a number of fitness QTLs underlying local adaptation (Ågren *et al.*, 2013). Laboratory experiments demonstrated higher freezing tolerance of the Swedish (SW) line and identified two QTLs of large effect that explained most of the parental difference in freezing tolerance (Oakley *et al.*, 2014). Both of these QTLs co-localized with QTLs for overall fitness, indicating that freezing tolerance contributes to local adaptation in these populations. Moreover, a transcription factor gene (*CBF2*), known to be a major regulator of freezing tolerance in response to cold acclimation (Thomashow, 1999, 2010), was demonstrated to be a causal gene underlying the largest effect freezing tolerance QTL (Gehan *et al.*, 2015). Other studies have shown that the SW line exhibits a greater up-regulation of, and has a higher photosynthetic capacity than, the Italian (IT) line when grown under cool temperature (Cohu *et al.*, 2013; Adams *et al.*, 2014; Stewart *et al.*, 2015). It has, furthermore, been suggested that the CBF locus may mediate crosstalk between cold acclimation and photosynthesis (Hüner *et al.*, 2012; Preston and Sandve, 2013; Stewart *et al.*, 2015).

Taken together, this system provides a unique opportunity to link adaptive differentiation to the genomic regions and physiological mechanisms that underlie responses to low temperature. In addition, we used recently developed protocols leveraging specialized growth chambers equipped with fluorescence imaging technology to obtain *in situ* whole-plant estimates of a number of photosynthesis-related parameters simultaneously (*F*_v_/*F*_m_, Φ_II_, NPQ, NPQ_fast_, NPQ_slow_, and qL; Table 1 and below) in a large number of plants (Cruz *et al.*, 2016), to provide insight into photosynthetic responses to low temperature. Chlorophyll fluorescence is a powerful approach for evaluating plant response to different environmental stresses (Maxwell and Johnson, 2000; Adams and Demmig-Adams, 2004; Baker, 2008), but only recently has it been possible to apply this technology at the scale needed for genetic mapping studies (Flood *et al.*, 2011). We ask the following questions. What is the genetic basis of photosynthetic responses to low temperature? To what extent do different photosynthetic traits have different or shared genetic bases, and what are some candidate genes underlying these QTLs? Do photosynthesis QTLs co-localize with fitness QTLs identified from field experiments at the parental sites?

**Table 1.** Photosynthetic parameters estimated and relationships between different parameters^a^

Parameter	Formula	Description
*F*_v_/*F*_m_	(*F*_m_–*F*_o_)/*F*_m_	Measure of maximum quantum efficiency of PSII in the dark-adapted state
Φ_II_	(*F*_m_'–*F*')/*F*_m_'	Measure of quantum efficiency of PSII under steady-state actinic light
NPQ	(*F*_m_–*F*_m_')/*F*_m_'	Non-photochemical quenching: the loss of maximal fluorescence from dark- to light-adapted states due to thermal dissipation
NPQ_fast_	*F*_m_/*F*_m_'–*F*_m_/*F*_m_''	Rapidly relaxing component of NPQ, attributable mainly to the qE processes
NPQ_slow_	(*F*_m_–*F*_m_'')/*F*_m_''	Slow relaxing component of NPQ, predominantly attributable to photoinhibition, but may also contain contributions from the xanthophyll cycle (qZ) chloroplast movements
qL	[(1+NPQ)/(1/Φ_II_−1)]/4	Estimates the fraction of open PSII centers^b^

*F*_o_, minimum fluorescence of a dark-adapted plant before actinic flash; *F*_m_, maximal fluorescence of a dark-adapted plant in a saturating actinic flash; *F*', fluorescence under steady-state light intensity before a asaturating actinic flash; *F*, _m_', maximal fluorescence under steady-state light intensity during a saturating actinic flash; *F*, _m_'', maximal fluorescence in darkness recovery from steady-state light intensity after the quick relaxing quenching has dissipated.

a Baker (2008) and Maxwell and Johnston (2000).

b The formula assumes maximal *F*_v_/*F*_m_=0.8.

## Materials and methods

### Creation of the RIL population

The RIL population (see Ågren *et al.*, 2013 for additional details) employed here was derived from a cross between a dam from the SW site and a sire from the IT site. The hybrid was selfed, and lines were propagated by single seed descent from autonomous selfing for nine generations. The resultant 544 RILs were genotyped using the Illumina Golden Gate Assay, and 348 single nucleotide polymorphisms (SNPs) were retained after quality filtering. A linkage map was constructed with these SNPs using the maximum likelihood algorithm and the Kosambi mapping function in JoinMap 4 (Van Ooijen, 2006). This resulted in a linkage map with an average marker spacing of ~1 cM, and ~1% missing data over the entire RIL population. Germplasm and genotype information for these RILs is publically available (ARBC: CS98760), as are the phenotypic data on fitness and freezing tolerance from Oakley *et al.* (2014) (Dryad: http://dx.doi.org/10.5061/dryad.h2c0c).

### Assay

We initiated the experiment with 506 RILs. Due to growth chamber space limitations, we tested these RILs in 11 batches (temporally separated experiments using the same growth chamber). Each batch contained 248 pots distributed across eight flats, with 32 replicates of each of the parental lines and four replicates of each of 46 RILs (unique to that batch). Plants were randomized in a stratified fashion such that three replicates of each parental line were assigned to each flat, and no flat contained more than one replicate of any of the RILs. For ~8–10% of pots (similar values for both parental lines and the RILs overall), seeds either failed to germinate or seedlings did not survive until the assay. To ensure that estimates of RIL phenotypes were averaged over any potential within-chamber variation, we pruned the data for RILs with <3 phenotyped replicates. We ran a 12th batch of the pruned RILs and some of the remaining 38 available RILs as described above, bringing the total number of RILs up to 495. In total, we phenotyped 1847 plants from the RILs, and ~350 plants from each parent.

Plants were grown and phenotyped at the Center for Advanced Algal and Plant Phenotyping (CAAPP) in the US DOE Plant Research Laboratory at Michigan State University using Dynamic Environmental Phenotyping Imaging (DEPI) chamber protocols (Cruz *et al.*, 2016). Each pot (5 cm square, 10 cm deep) was filled with Sunshine Redi-Earth, treated with Gnatrol fungicide, and watered. Pots were then covered with a low reflectance black, foam mask with a hole in the center, into which 2–3 seeds were sown. Flats were covered and stratified at 6 °C in the dark for 3 d to break dormancy and synchronize germination. Flats were then removed from stratification and placed in growth chambers set for 8 h days at 21 °C with a light intensity of 100 µmol photons m^−2^ s^−1^. Once seeds had germinated, we thinned the pots, keeping the central-most seedling. Flats were bottom watered three times per week, twice with deionized water, and once with CAAPP nutrient solution (Lu *et al.*, 2011*b*) until plants were 24 d old. To prepare for DEPI conditions, light intensity was increased to 500 µmol photons m^−2^ s^−1^ before transferring plants. Plants were then transferred into the DEPI chamber at 21 °C, the light intensity was increased to 800 µmol photons m^−2^ s^−1^, and plants were allowed to acclimate for 24 h before the start of the DEPI imaging protocol. The DEPI protocol began with 1 d at 21 °C, 800 µmol photons m^−2^ s^−1^ (day 0) then, immediately after the second measurement of *F*_v_/*F*_m_ (day 1), temperature was reduced to 4 °C at the same light intensity for the remaining days. Eight hour days were maintained throughout the DEPI experiment (days 2–8). In both parental field locations, plants experience cold temperatures and short-day conditions similar to what we impose here (Dittmar *et al.*, 2014). While the results from our assay are likely to vary with different combinations of temperature, photoperiod, and PAR, our study is a first step towards understanding the genetic basis of photosynthetic responses to one ecologically relevant set of conditions using these new techniques.

**Fig. 1.**
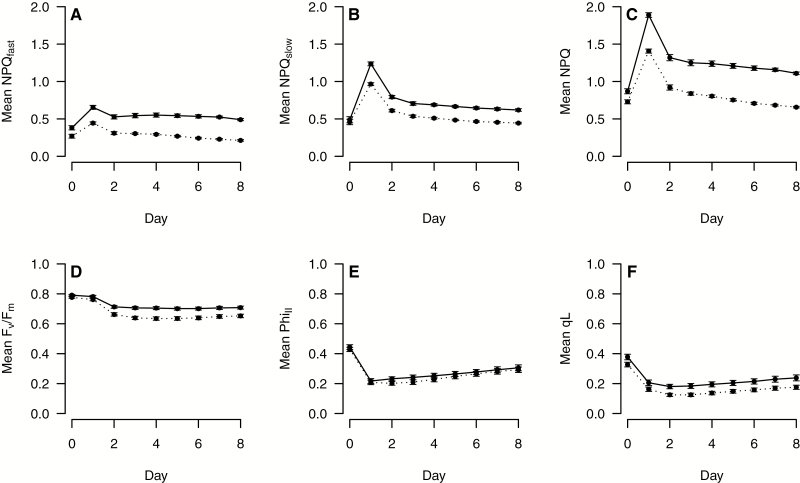
Parental daily means for six photosynthetic traits (see Table 1 for an explanation of trait abbreviations) over the course of the experiment (Italian, dashed line; Swedish, solid line). Day 0 measurements were taken at 21 °C prior to the start of the cold treatment; the remaining days were all 4 °C. Means were calculated by flat within batch, and then over batches. Error bars are 1 SE.

We estimated several photosynthesis-related parameters (Table 1) using pulse-amplitude modulation (PAM) chlorophyll fluorescence methods modified for fluorescence imaging (Cruz *et al.*, 2016). For full explanations of these parameters, see the review by Baker (2008). The maximum PSII quantum efficiency in dark-adapted leaves was estimated by *F*_v_/*F*_m_ once per day prior to the first illumination in the morning. The steady-state PSII quantum efficiency (ϕ_II_) and the total NPQ and its components, NPQ_fast_ (largely reflecting qE) and NPQ_slow_ (largely reflecting a combination of qI, but see caveat below), were estimated hourly during the illumination period following Cruz *et al.* (2016). Measurements were not taken more often because preliminary results indicated that increasing the frequency of the saturating light pulses needed for PAM substantially inhibited Arabidopsis plants at low temperatures. The dark time prior to measurement of *F*_m_'' was set at 2 min, which was found to allow for the majority of rapid NPQ relaxation, while not affecting the slower components, and because a longer dark exposure was found to induce separate stress responses (Cruz *et al.*, 2016). However, it is important to note that with NPQ_slow_, we did not distinguish between true photoinhibition (qI) and other processes, such as qZ, a quenching process related to the accumulation of zeaxanthin (Demmig-Adams *et al.*, 2012, 2014; Sylak-Glassman *et al.*, 2014). We also do not distinguish NPQ_slow_ from state transitions that could contribute to longer-lived NPQ signals, though these are unlikely to be important except under very low light (Mullineaux and Emlyn-Jones, 2005). Thus, we designate our NPQ parameters as NPQ_fast_ and NPQ_slow_ for the rapidly and slowly relaxing forms. For all traits other than *F*_v_/*F*_m_, we averaged the hourly measurements for an individual plant to produce a single daily mean, giving a single estimate for each plant on each day. Daily means for *F*_v_/*F*_m_ and ϕ_II_ were used to calculate qL, a parameter that reflects the redox state of Q_A_, the primary quinone electron carrier in PSII (Kramer *et al.*, 2004). In three separate batches, there were technical issues that resulted in missing data for 1 d (days 4, 7, and 8 respectively). We therefore used the mean values of days 5 and 6 for all subsequent analyses because there were stable differences between the parents over this duration (Fig. 1), and because it allowed us to maximize the number of RILs in our QTL analyses.

### Statistical analyses

Least square means (LSMs) for each of the RILs and parental lines were calculated separately for each trait using the raw mean values of individual plants (days 5 and 6) as the dependent variable. Model effects included line (each RIL plus the two parental lines) as a fixed effect and batch and flat nested within batch as random effects. Models were fit using Proc Mixed in SAS v9.3 (SAS, 2011). For each trait, LSM comparisons of the parental means were used to evaluate significant differences between the parents, and LSMs for the RILs were used in subsequent QTL analyses.

Some traits were excluded from QTL analyses because parental differences were small relative to variation among batches. We expect that adaptive traits involved in local adaptation will be subject to divergent selection in different populations, thus our primary focus is on traits with marked differences between the parents. In addition, our experimental design will be sensitive to among-batch variation, particularly with limited differences between the parents. We therefore used a preliminary ANOVA on the parental values only, treating both genotype and batch as random effects to compare variance components of these two effects. Virtually all of the variation in ϕ_II_ was due to batch (81% compared with 3% for genotype), so it was excluded. We likewise excluded qL because the variation among batches (54%) greatly exceeded variation due to genotype (33%). Variation among batches was substantial for *F*_v_/*F*_m_ (36%), but we included this trait because the variation due to genotype was larger (54%). For all the remaining traits (NPQ, NPQ_fast_, and NPQ_slow_), genotype explained >73% of the variation, and batch explained <9%.

We performed QTL analyses using R/qtl (Broman and Sen, 2009) following procedures previously employed for this study system (Ågren *et al.*, 2013, 2017; Dittmar *et al.*, 2014; Oakley *et al.*, 2014; Postma and Ågren, 2016). In brief, for each trait we quantile normalized the data (Broman and Sen, 2009) and determined the best multiple QTL model using Haley–Knott regression based on genotype probabilities and filling in gaps >2 cM with pseudomarkers. We used the automated stepwise model selection procedure (Manichaikul *et al.*, 2009), and scanned for additive QTLs and digenic interactions at each step using logarithm of the odds (LOD) thresholds determined from 10 000 permutations with an experiment-wise α=0.05. For each QTL, we calculated Bayesian 95% credible intervals around the point estimates and used the fitqtl function (Broman and Sen, 2009) to calculate the percentage variance explained (PVE). Finally, we refit these QTL models with the non-normalized data to generate genotypic effect sizes for each QTL in units of the individual traits. Model selection for QTLs for NPQ (not shown) produced a QTL model that was not representative of the QTL models of its component parts NPQ_fast_ and NPQ_slow_, so we report QTLs for NPQ_fast_ and NPQ_slow_ separately and omit results for NPQ.

### Co-localization of photosynthesis QTLs and candidate genes

We examined co-localization between QTLs for different photosynthetic traits by comparing overlap of the 95% credible intervals of each QTL. We considered QTLs to be the same if their credible intervals overlapped, and different if they did not. We also examined the genes within our Psyn QTL with Credible Intervals smaller than 15.2 cM (a quarter of the length of the smallest chromosome), as in previous studies (Dittmar *et al.*, 2014; Oakley *et al.*, 2014). To identify possible candidate genes that underlie QTLs, we filtered the list of all genes under each QTL following two criteria based on parental data. We identified genes with *in silico* predictions of amino acid differences (including premature stop codons) between the parents (J.K. McKay, unpublished data). We also used a previously published RNAseq data set [(Gehan *et al.*, 2015); GEO: GSE67332] to identify genes that are differentially expressed [false discovery rate (FDR)=0.01 and log_2_ fold change >1] between the two lines after 1 week at 4 °C (conditions similar to the 5–6 d at 4 °C plants experienced in the present study) compared with warm temperatures.

### Co-localization of photosynthesis QTLs with fitness QTLs

To determine if the QTLs identified for the measured photosynthetic traits might contribute to fitness in nature, we examined co-localization between individual photosynthesis QTLs and previously published fitness QTLs derived from field studies carried out over 3 years at both parental sites (Ågren *et al.*, 2013). Our criterion for co-localization was that the point estimate of an individual photosynthesis trait QTL that could be assigned to a relatively narrow genomic region (with a credible interval <15.2 cM) must be within the range of point estimates of fitness QTLs that were detected in more than one site and/or year.

## Results

### Parental means and RIL distributions

**Fig. 2.**
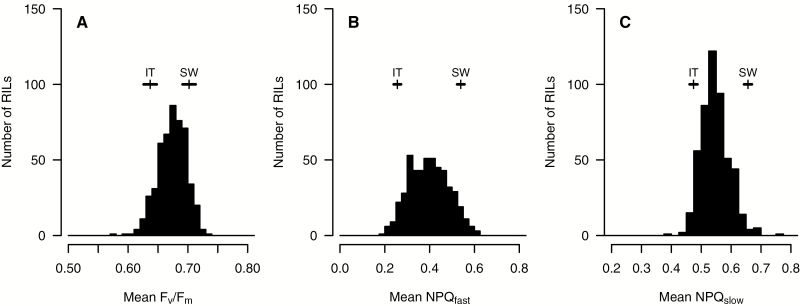
Frequency distributions of recombinant inbred line (RIL) means for each of three photosynthetic traits in the QTL analyses: (A) maximal quantum efficiency of PSII in the dark-adapted state, *F*_v_/*F*_m_; (B) fast relaxing non-photochemical quenching, NPQ_fast_; and (C) slow relaxing non-photochemical quenching, NPQ_slow_. Note the difference in the *x*-axis scale between different traits. For each trait, means (vertical tick) and 1 SE (horizontal bar) are given for the Italian (IT) and Swedish (SW) parents. Least square mean comparisons between the parents are significant (*P*<0.001) for all three traits.

For most of the six estimated traits (NPQ_fast_, NPQ_slow_, NPQ, *F*_v_/*F*_m_, Φ_II_, and qL; Fig. 1), parental mean values were very similar in the initial, warm environment (21 °C, day 0; Fig. 1), but SW and IT lines differed in their responses to cold. Initiation of the cold treatment on day 1 resulted in transient spikes in NPQ and its components NPQ_fast_ and NPQ_slow_ (Fig. 1A–C) that were greater in the SW line than in the IT line. These quenching parameters recovered partially on the following day, but remained higher in the SW line compared with the IT line for the duration of the cold period (Fig. 1). For *F*_v_/*F*_m_, Φ_II_, and qL, trait values decreased in response to the initial days of cold, followed by a leveling (*F*_v_/*F*_m_) or a gradual recovery (Φ_II_ and qL) over the course of several days (Fig. 1D–F). The parental lines had nearly parallel responses for *F*_v_/*F*_m_ and qL (SW greater than IT), and parental means for Φ_II_ were very similar over the course of the experiment. For the mean values of days 5 and 6 in the cold, there were highly significant differences between the parental values for all six traits (*P*<0.001; Fig. 2; Supplementary Fig. S1 at *JXB* online). For the three traits in the QTL analyses, the SW line had 9% (*F*_v_/*F*_m_), 52% (NPQ_fast_), and 39% (NPQ_slow_) higher LSM trait values than the IT line, and the RIL means were largely intermediate to the parental trait values (Fig. 2).

### Mapping *F*_v_/*F*_m_

**Fig. 3.**
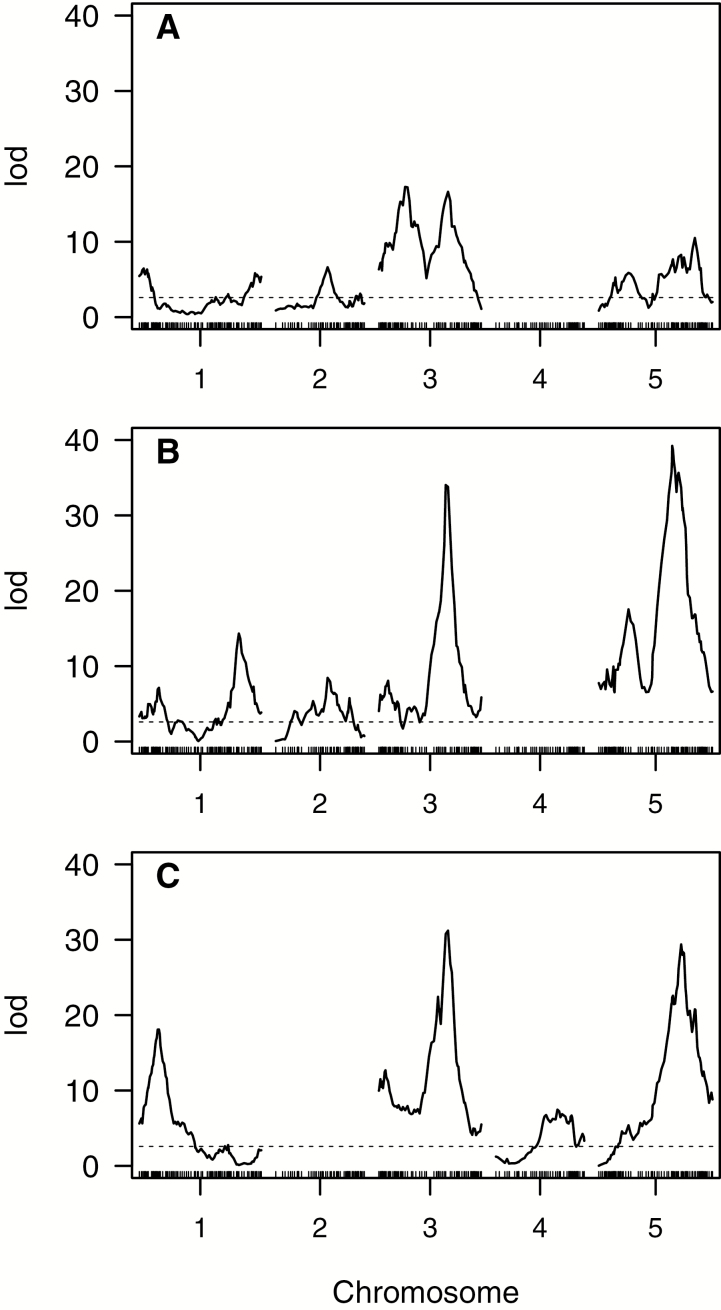
Multiple QTL model logarithm of the odds (LOD) profile plots for: (A) maximal quantum efficiency of PSII in the dark-adapted state, *F*_v_/*F*_m_; (B) fast relaxing non-photochemical quenching, NPQ_fast_; and (C) slow relaxing non-photochemical quenching, NPQ_slow_. Multivariate LOD profiles here are a measure (log scale) of association between marker genotype and phenotype. The significance threshold based on 10 000 permutations is given by the dashed lines.

The full QTL model for *F*_v_/*F*_m_ included nine QTLs (Fig. 3A; Supplementary Table S1) explaining 55.0% of the total variation (PVE). The SW genotype increased the trait value for six of the nine QTLs. For the QTLs with positive effects of the SW genotype, the average LOD score and PVE were 9.97 (range=2.98–17.28) and 4.43 (range=1.27–7.85), respectively (Supplementary Table S1). For the QTLs with negative effects of the SW genotype, the average LOD score and PVE were 5.15 (range=3.11–6.45) and 2.21 (range=1.32–2.78), respectively (Supplementary Table S1).

**Fig. 4.**
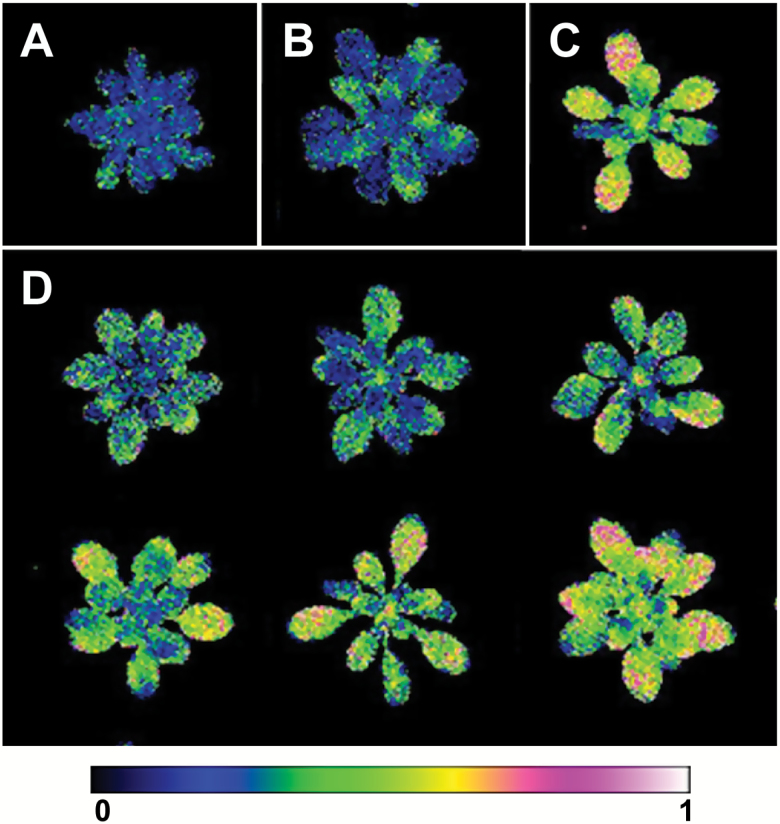
False-color images of fast relaxing non-photochemical quenching (NPQ_fast_) after 6 d of cold treatment. (A) Italian parent; (C) Swedish parent; (B) IT×SW F_1_ cross; and (D) six RILs representative of the range of phenotypes observed.

### Mapping NPQ_fast_

Of the three traits that we mapped, parental differences in NPQ_fast_ were the most striking (Figs 3B, 4A, C). The full QTL model for NPQ_fast_ included 13 QTLs and one epistatic interaction (Supplementary Fig. S2B; Supplementary Table S1) and had a total PVE of 64.9%. For the individual QTL effects, the SW genotype increased the trait value for 9 of the 13 QTLs. For the QTLs with positive effects of the SW genotype, the average LOD score and PVE were 13.22 (range=2.79–39.22) and 4.89 (range=0.92–15.46), respectively (Supplementary Table S1). For the QTLs with negative effects of the SW genotype, the average LOD score and PVE were 10.77 (range=5.36–17.53) and 3.75 (range=1.80–6.22), respectively (Supplementary Table S1). The two QTLs with the largest negative effect sizes, Psyn1:5 and Psyn5:2, were also identified in the multiple QTL model as having a significant epistatic interaction (LOD=5.90).

### Mapping NPQ_slow_

The full QTL model for NPQ_slow_ was composed of eight QTLs (Fig. 3C; Supplementary Table S1) and had a total PVE of 53.5%. The SW genotype increased trait values for six of the eight QTLs. No epistatic interactions met the significance thresholds of the model selection process, though there appears to be an interaction between chromosome 3 and chromosome 5 that may be near the threshold (Supplementary Fig. S2C). For the QTLs with positive effects of the SW genotype, the average LOD score and PVE were 16.93 (range=2.76–31.20) and 8.20 (range=1.21–15.66), respectively (Supplementary Table S1). For the QTLs with negative effects of the SW genotype, the average LOD score and PVE were 5.44 (range=5.38–5.50) and 2.41 (range=2.39–2.44), respectively (Supplementary Table S1).

### Co-localization of individual trait QTLs

**Fig. 5.**
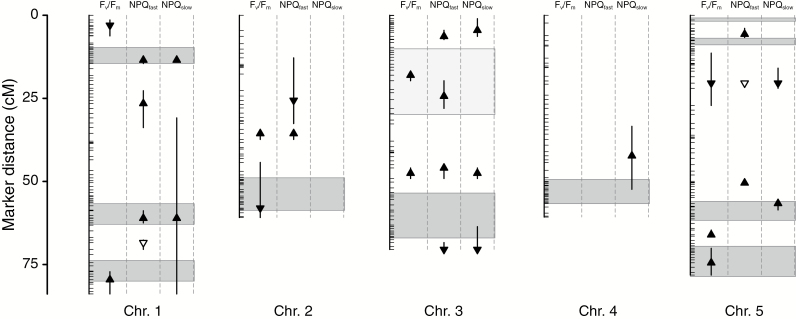
Photosynthesis QTLs detected in the present study and fitness QTLs detected in the field (from Ågren *et al.*, 2013). Vertical lines with horizontal tick marks are the five chromosomes (Chr.) and marker positions. Distances along the chromosomes are given on the *y*-axis in centiMorgans (cM). Triangles represent point estimates of the location of photosynthesis QTLs (maximal quantum efficiency of PSII in the dark-adapted state, *F*_v_/*F*_m_; fast relaxing non-photochemical quenching, NPQ_fast_; and slow relaxing non-photochemical quenching, NPQ_slow_), and the direction of the arrow indicates the effect of a substitution of the Swedish genotype (upwards=greater trait value, downwards=lesser trait value). The two open triangles are QTLs involved in an epistatic interaction for NPQ_fast_. Vertical lines for each QTL are the Bayesian 95% credible intervals around the point estimate. Shaded boxes represent the range of point estimates of fitness QTLs from field studies (from Ågren *et al.*, 2013) where QTLs were detected in more than one year/site (darker boxes=fitness QTLs detected in both parental sites; the single lighter shaded box was detected in multiple years at the Swedish site only).

Based on the criteria of overlapping QTL credible intervals, we were able unambiguously to assign the 30 individual trait QTLs to 20 unique photosynthesis (Psyn) QTLs (Supplementary Table S1). There were two exceptions. We assigned NPQ_slow_ at position 61.1 to Psyn1:4 despite its very wide credible interval because it shared the same point estimate as the QTLs for NPQ_fast_ (Supplementary Table S1). For Psyn3:2, the opposite ends of the credible intervals for QTLs for NPQ_fast_ and *F*_v_/*F*_m_ share a single marker in common. A likelihood ratio test comparing a two QTL model to a single QTL model, using the sum of the LOD scores of the two QTLs and the peak LOD score of the summed LOD profiles (Leinonen *et al.*, 2013; Oakley *et al.*, 2014), gave no support for these being distinct QTLs (χ^2^=0.39, 1 df, *P*=0.53), so we consider them to be the same. Of the individual QTLs for each trait, 56, 38, and 25% of QTLs for *F*_v_/*F*_m_, NPQ_fast_, and NPQ_slow_, respectively, were unique to that particular trait (no overlap) and thus exhibited clearly distinct genetic bases. These ‘singleton’ QTLs included one major QTL each for NPQ_fast_ (Psyn5:3), NPQ_slow_ (Psyn5:4), and *F*_v_/*F*_m_ (Psyn5:5). Psyn5:3 (NPQ_fast_, LOD=39.2) and Psyn5:4 (NPQ_slow_, LOD=29.4) were only 6.1 cM apart, but are clearly distinct based on their credible intervals (Fig. 5; Supplementary Table S1).

There were eight Psyn QTL represented by more than two trait QTLs. For the three possible combinations of overlapping QTLs for two traits, the most common (four) was NPQ_fast_ and NPQ_slow_. There were two cases of overlap of *F*_v_/*F*_m_ and NPQ_fast_, and no overlapping QTLs for *F*_v_/*F*_m_ and NPQ_slow_. There were two cases where QTLs for all three traits overlapped, one of which (Psyn3:3) was a major effect QTL for all three traits (Fig. 5; Supplementary Table S1). The other case (Psyn5:2) was at the location of one of the epistatic QTLs for NPQ_fast_; despite the absence of epistatic interactions for either *F*_v_/*F*_m_ or NPQ_slow_, both traits also had QTLs at this location.

### Candidate genes under QTLs

There were 17 Psyn QTLs for which at least one trait QTL had a credible interval <15.2 cM (Supplementary Table S1) that were included in the candidate gene search. On average, there were 276 genes (range=94–560) with either (or both) predicted amino acid differences or gene expression differences in response to cold between SW and IT under these QTLs. For the QTLs with the largest effect sizes (Psyn3:2, Psyn3:3, Psyn5:3, Psyn5:4, and Psyn5:5) there were 287, 79, 107, 185, and 151 genes, respectively, matching the above criteria. Some notable genes under our QTLs include some with previously recorded chlorophyll fluorescence phenotypes including *LQY1* (Lu *et al.*, 2011*a*) under Psyn1:6 for *F*_v_/*F*_m_, and *NPQ6* (Jung and Niyogi, 2010) under Psyn5:3 for NPQ_fast_, as well as *NPQ2* (under Psyn5:6 for *F*_v_/*F*_m_), which is important for recovery from quenching (Kromdijk *et al.*, 2016). While we cannot rule out any genes within the QTL credible intervals as causal, these candidates represent the highest priority for future functional studies. We can, however, rule out some genes well known to be important for photosynthesis, such as *NPQ1* and *NPQ4* (reviewed in Brooks *et al.*, 2014), because these do not occur within any of our QTLs.

### Co-localization with fitness QTLs

There were seven Psyn QTLs with reasonably small credible intervals that overlapped with fitness QTLs (Ågren *et al.*, 2013) observed over multiple years and/or sites (Fig. 5; Supplementary Table S1). Six of these seven occurred in regions of the genome where the local genotype was consistently favored in at least one environment (Ågren *et al.*, 2013), and are therefore potentially adaptive. Three of the potentially adaptive photosynthesis QTLs (Psyn1:6, Psyn2:2, and Psyn5:6; all *F*_v_/*F*_m_ only) overlapped with different fitness trade-off QTLs (Ågren *et al.*, 2013). For the remaining three photosynthesis QTLs associated with fitness QTLs (Psyn1:2 and 1:4 involving both NPQ_fast_ and NPQ_slow_, and Psyn5:4, a major QTL for NPQ_slow_), the IT genotype was favored at both sites (Ågren *et al.*, 2013).

## Discussion

### Parental differences in response to cold

The parental lines in this study are adapted to dramatically different climates (Ågren and Schemske, 2012; Ågren *et al.*, 2013; Dittmar *et al.*, 2014; Oakley *et al.*, 2014), but in both locations, ~1-month-old plants experience a combination of temperature, day length, and PAR similar to experimental conditions imposed here (4 °C, 8 h days, 800 PAR). At the initial warm temperature (21 °C) imposed in our study, the parental lines exhibited similar values of all parameters measured (Fig. 1), indicating that the differences observed and mapped at 4 °C represent genetically based differences in physiological responses to low temperature.

At 4 °C, the SW line had greater values than the IT line for all parameters except Φ_II_ (Fig. 1). These differences were most pronounced and consistent for *F*_v_/*F*_m_ and NPQ, and components NPQ_fast_ and NPQ_slow_ (Fig. 2). Greater NPQ indicates that the SW line has a greater capacity to dissipate excess light energy as heat, and that this appears to occur through both rapid (NPQ_fast_) and slow relaxing mechanisms (NPQ_slow_ and qZ; Table 1), suggesting that both play a role in the SW line in protecting photosynthesis at low temperatures.

Greater *F*_v_/*F*_m_ for the SW line indicates a greater maximal quantum efficiency of photosynthesis in the dark-adapted (i.e. pre-dawn) state, most probably reflecting an ability to maintain a higher level of active PSII centers. This may come about by greater capacity for photoprotection, as reflected in the higher NPQ extents during the day.

Another mechanism by which the SW line might more effectively reduce excitation pressure (Hüner *et al.*, 2012) is through up-regulation of photosynthetic capacity facilitated by greater export of foliar carbohydrates to sinks elsewhere in the plant (Adams *et al.*, 2013). Previous work has shown that in response to a regime of 8 °C days/12.5 °C nights (compared with 25 °C days/20 °C nights), the SW line (in comparison with the IT line) exhibited anatomical and ultrastructural changes resulting in an increased photosynthetic capacity via a greater ability to export foliar carbohydrates (Cohu *et al.*, 2013; Adams *et al.*, 2014; Demmig-Adams *et al.*, 2014; Stewart *et al.*, 2015). Unfortunately, such measurements are not amenable to high-throughput methods as employed in our QTL mapping study. In addition, it is difficult to extrapolate from a single cold condition used previously (8 °C days/12.5 °C nights) to the single cold condition used in our study (4 °C) because there is some evidence for non-linear responses of photosynthetically related traits as a function of temperature (Hetherington *et al.*, 1989; Hüner *et al.*, 1993). Other methodological differences make it difficult to compare results, such as smaller pot sizes in our experiment which could limit the capacity of roots to serve as a sink for carbohydrates, though plants in our experiment are comparable in size to field grown plants.

### Mapping *F*_v_/*F*_m_

In many species, *F*_v_/*F*_m_ in unstressed plants is consistently ~0.83 (Baker, 2008), and typically decreases after stress. In the present study, greater *F*_v_/*F*_m_ in the SW line is therefore consistent with increased photosynthetic acclimation in response to low temperature. QTLs with positive effects (the SW genotype increases trait value) can contribute to these putatively adaptive differences. Six of the nine QTLs for *F*_v_/*F*_m_ had positive effects. For this and other traits, the direction of effect of the major QTLs (≥20% of the difference in parental values) is of particular interest. For *F*_v_/*F*_m_, all of the major QTLs (Psyn3:2, Psyn3:3, and Psyn5:5; Fig. 3; Supplementary Table S1) had positive effects. Major effect QTLs in the direction of the parental differences may be responsible for large-scale divergence (i.e. opposing directional selection between two distant phenotypic optima) (Orr, 1998*a*; Dittmar *et al.*, 2016), while smaller effect QTLs with a mixture of positive and negative effects might indicate a history of stabilizing selection around local optima (Dittmar *et al.*, 2016). This is one possible example of a scenario where not all adaptive QTLs would be expected to have the same sign (cf. Orr, 1998*b*).

Some studies have quantified natural variation in *F*_v_/*F*_m_ in Arabidopsis. For example, Zhen *et al.* (2011) compared *F*_v_/*F*_m_ in accessions from the northern and southern portion of the range after acclimation for 3 d at 4 °C. Surprisingly, they did not find significant regional differences in *F*_v_/*F*_m_. We note that our SW population comes from a location ~7 ° further north than the northernmost accessions in Zhen *et al.* (2011), but other methodological differences might also explain our different results.

To our knowledge, ours is the first study in Arabidopsis to examine the genetic basis of *F*_v_/*F*_m_, albeit in a single set of environmental conditions. Comparable studies in other species are rare. Heo *et al.* (2014) conducted QTL mapping of *F*_v_/*F*_m_ in *Boechera stricta* in multiple controlled environments and in the field, and for the conditions most similar to the present study (short days and cold acclimated; SDCA), found a QTL for *F*_v_/*F*_m_ in a region syntenic with the Arabidopsis CBF regulon, a major regulator of freezing tolerance (Thomashow, 2010). However, parental values of *F*_v_/*F*_m_ were identical in the SDCA environment, so it is unclear how this QTL could contribute to population ifferentiation for this trait.

### Mapping NPQ_fast_ and NPQ_slow_

Greater NPQ in SW in response to cold suggests that SW has a more effective mechanism for dissipating excess light energy as heat. Greater NPQ_fast_ in the SW line compared with the IT line means that energy-dependent processes contribute to overall differences in NPQ, and therefore QTLs with positive effects (the SW genotype increases trait value) on NPQ_fast_ contribute to observed differentiation in NPQ_fast_ and overall NPQ. The pattern for NPQ_slow_ was similar to that for NPQ_fast_, but the explicit mechanism of quenching via NPQ_slow_ could not be determined from the present data (some combination of qI and qZ). Nine of the 13 QTLs for NPQ_fast_ had positive effects, including the two major QTLs for NPQ_fast_ (Psyn3:3 and Psyn5:3), with large effect QTLs for NPQ_slow_ (Psyn3:3 and Psyn5:4) also having positive effects. Previous work has mapped the genetic basis of NPQ in Arabidopsis in response to high light (which can produce similar excitation pressure to lower light in cold temperatures), though it did not distinguish between NPQ_fast_ and NPQ_slow_ (Jung and Niyogi, 2009). The authors identified two QTLs, one of which was near two of the QTLs found in our study: Psyn1:2 (NPQ_fast_ and NPQ_slow_) and Psyn1:3 (NPQ_fast_).

### Co-localization of individual photosynthesis trait QTLs

We assigned the 30 individual trait QTLs to 20 unique Psyn QTLs. Only detailed genetic studies can determine if the Psyn QTLs we have identified represent single genes that control multiple traits. Nevertheless, two lines of evidence suggest a shared genetic basis. First, most co-localizing QTLs had identical point estimates. Secondly, all groups of co-localized QTLs had the same direction of effects. Psyn3:3 is a striking example of a shared genetic basis for multiple traits because it contains a major QTL for all three traits. A previous study in barley provides some evidence for co-localization of QTLs between *F*_v_/*F*_m_ and NPQ (Tyrka *et al.*, 2015). This result might be expected because of the potential mechanistic link between these traits. For instance, a gene that increases photoprotection through NPQ during the day (via either NPQ_fast_ or NPQ_slow_) would be expected to reduce photodamage, and thus the NPQ components that tend to depress *F*_v_/*F*_m_. The most common pairwise co-localization of trait QTLs in the present study was between NPQ_fast_ and NPQ_slow_. Interestingly, recent work (Davis *et al.*, 2016) demonstrated a functional link between qE and qI involving the thylakoid proton motive force (pmf). The pmf, which is generated by the light-driven electron and proton transfer reactions in the thylakoids, served both to drive the synthesis of ATP and, by its effects on lumen pH, to activate the qE response. High levels of pmf (in particular, the electric field component) also induce back-reactions within the PSII reaction centers that can generate singlet oxygen and induce PSII inactivation and qE. It is thus conceivable that these loci affect the thylakoid pmf at low temperatures, a proposal that will be tested in future work.

Despite overall evidence of a common shared genetic basis, for each trait there was a major effect QTL that was unique to that trait; that is, it did not co-localize with other QTLs (Psyn5:3 for NPQ_fast_, Psyn5:4 for NPQ_slow_, and Psyn5:5 for *F*_v_/*F*_m_). The shared QTL Psyn3:3 and the large effect QTLs unique to each trait merit further study (using near isogenic lines grown in multiple environments) for disentangling the genetic basis of natural variation in photosynthetic responses to cold.

### Candidate genes

For each QTL identified in our study, there are of the order of 100 genes or more predicted to have non-synonymous substitutions and/or are differentially expressed in response to 4 °C. Future functional work is needed to narrow down and validate these candidates, but we briefly discuss a few example genes of particular interest. The CBF locus has been hypothesized to condition photosynthetic acclimation to cold (Dahal *et al.*, 2012; Hüner *et al.*, 2012; Preston and Sandve, 2013; Demmig-Adams *et al.*, 2014), and there is evidence that *CBF2* is the causal gene underlying a major QTL that contributes to the difference in freezing tolerance between the SW and IT lines (Oakley *et al.*, 2014; Gehan *et al.*, 2015). However, with the exception of one relatively weak QTL for NPQ_slow_, we found no photosynthesis QTLs near the CBF locus (cf. Heo *et al.*, 2014). Also, notably absent within our QTLs were genes known to be important in NPQ_fast_, such as *NPQ4* and *NPQ1* (Brooks *et al.*, 2014). The lack of a QTL at *NPQ4*, which encodes the PsbS protein, an essential component for qE, lends further support to the idea that natural variation in non-photochemical quenching in Arabidopsis is not based on variation in PsbS (Jung and Niyogi, 2009; Johnson and Ruban, 2010).

Genes of particular interest that were within our QTLs include those with effects on non-photochemical quenching such as *NPQ6* (Jung and Niyogi, 2010), as well as *NPQ2*, which has recently been shown to influence yield in transgenic tobacco (Kromdijk *et al.*, 2016). While we cannot conclude that these genes are causal in this RIL population, they do represent high priority candidates for future functional work. It is further notable that there were no strong candidates under Psyn3:3, which was the location of a major QTL for all three traits. Future work on this region may offer a unique opportunity to discover unknown genes that contribute to natural variation in photosynthetic responses.

### Co-localization with fitness QTLs

Any trait QTL that co-localizes with a fitness QTL could in principle be an adaptive trait QTL. Perhaps the most convincing evidence for such an adaptive trait QTL is a case where the trait QTL contributes to observed parental differences (i.e. the SW genotype at the QTL increases the trait value), and occurs in the same location as a fitness QTL (Ågren *et al.*, 2013) where the local genotype is favored in at least one environment. We find six such cases, and three of these (all *F*_v_/*F*_m_) co-localized with genetic trade-off QTLs (Ågren *et al.*, 2013). This suggests that *F*_v_/*F*_m_ in general, and these QTLs in particular, might contribute to fitness trade-offs across environments. However, for one of these three (Psyn2:2), the direction of effect of the QTL for *F*_v_/*F*_m_ was opposite to the parental differences, making its role in fitness trade-offs suspect. For the remaining three cases (two QTLs for both NPQ_fast_ and NPQ_slow_ and one QTL for just NPQ_slow_), the IT genotype was favored at both sites (Ågren *et al.*, 2013), which is difficult to explain in the context of adaptive differentiation for these traits.

An important caveat to our work is that our QTLs come from a single, albeit ecologically relevant, set of environmental conditions. Photosynthetic measurements under multiple conditions are necessary to make general conclusions about photosynthetic responses to cold, and would be useful for relating important photosynthesis QTLs to fitness QTLs. However, the mapping of almost 500 RILs with replication made testing in multiple environments unfeasible. Further experiments conducted in conditions simulating the parental environments with near isogenic lines for major Psyn QTLs are needed to understand how photosynthetic traits and their relationship to plant fitness change across environments.

## Supplementary data

Supplementary data are available at *JXB* online.

Table S1. Locations and effect sizes of photosynthesis QTLs.

Table S2. Candidate genes for photosynthesis QTLs.

Fig. S1. Frequency distributions of least square means (LSMs) for the RILs for the three photosynthetic traits not in the QTL analyses: (A) NPQ, (B) Φ_II_, and (C) qL.

Fig. S2. Heat maps of all pairwise additive and digenic interactions for (A) *F*_v_/*F*_m_, (B) NPQ_fast_, and (C) NPQ_slow_.

Supplementary Figures S1-S2 and Table_S1

Supplementary Table S2
