# Supplementary material for: Genetic basis of photosynthetic responses to cold in two locally adapted populations of Arabidopsis thaliana
Source: J Exp Bot. 2017 Dec 28;69(3):699–709. doi: 10.1093/jxb/erx437 (PMC5853396; doi:10.1093/jxb/erx437)
Supplement: Supplementary Figures S1-S2 and Table_S1 [file erx437_suppl_supplementary_figures_s1-s2_table_s1.pdf]

SUPPLEMENTARY INFORMATION for:

Genetic basis of photosynthetic responses to low temperature in two locally adapted populations of Arabidopsis thaliana

Christopher G. Oakley[1], Linda Savage[2], Samuel Lotz[2,3], G. Rudd Larson[2,4], Michael F. Thomashow[2,5], David M. Kramer[1,2,3], Douglas W. Schemske[1,6]

[1] Department of Plant Biology

[2] MSU-DOE Plant Research Laboratory

[3] Department of Biochemistry and Molecular Biology

[4] Genetics Graduate Program

[5] Plant Resilience Institute

[6] W. K. Kellogg Biological Station

Michigan State University, East Lansing, MI 48825, USA

Author for correspondence: Christopher G. Oakley

Present address: Department of Botany and Plant Pathology, and the Purdue Center for Plant Biology, Purdue University, West Lafayette, IN 47907-2054, USA; Telephone: 1-765-494-9644; E-mail oakleyc@purdue.edu

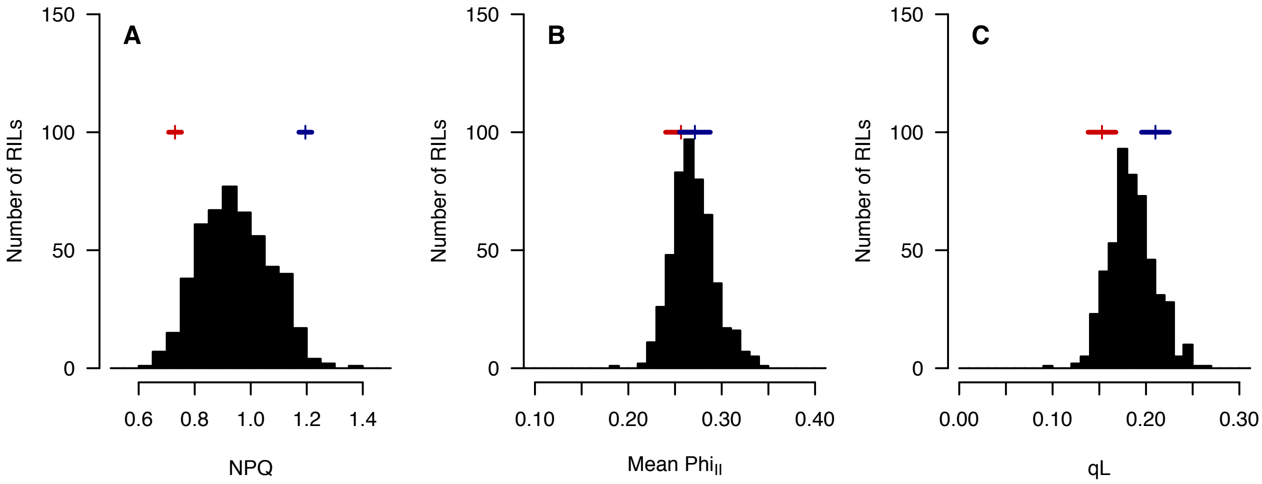

Fig. S1. Frequency distributions of Least Square Means (LSMs) for the recombinant inbred lines (RILs) for the three photosynthetic traits not in the QTL analyses: (A) NPQ – Non-Photochemical Quenching, (B) Φ_{II} – Steady-state quantum efficiency of photosystem II, and (C) qL – Fraction of open photosystem II centers. Note difference of x-axis scale between different traits. For each trait, means (vertical tick) and one standard error (horizontal bar) are given for the Italian (red) and Swedish (blue) parents. LSM comparisons between the parents are significant ($P < 0.001$) for all three traits, though differences between parents for Φ_{II} are small compared to the variation among the RILs.

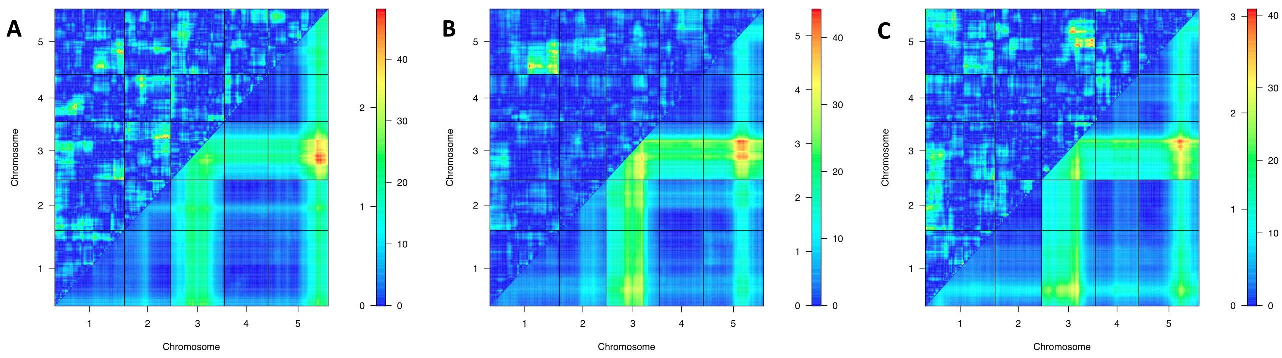

Fig. S2. Heat maps of all pairwise additive and digenic interactions for (A) Maximal quantum effiency of photosystem II in the dark adapted state - F_v/F_m, (B) Fast relaxing Non-Photochemical Quenching - NPQ_{fast}, and (C) Slow relaxing Non-Photochemical Quenching - NPQ_{slow}), produced using the scantwo function in R/qtl (Broman and Sen 2009). The LOD score of the full model (QTLi + QTLj + QTLi x QTLj) of every pairwise marker combination is plotted below the diagonal. The logarithm of the odds (LOD) scores above the diagonal correspond to digenic interactions (QTLi x QTLj). Vertical bars to the right of each pane are heat map look-up tables and give the full and interaction LOD scores on the right and left side, respectively.

Table S1. Locations and effect sizes of photosynthesis QTL (Fv/Fm = maximal quantum effiency of photosystem II in the dark adapted state, NPQfast = fast relaxing Non-Photochemical Quenching, and NPQslow = slow relaxing Non-Photochemical Quenching). Positions are given by Chromosome (Chr.) and position in cM (Pos.), and the lower and upper limits of the 95% Bayesian credible intervals (LCI and UCI, respectively). For each QTL, the logarithm of the odds (LOD) score, percent variance explained (PVE), and the effect of a substitution of the Swedish genotype in units of the trait value (SW2a) and standard error are given.

QTL	Trait	Chr.	Pos.	LCI	UCI	LOD	PVE	SW2a	SW2a SE	Epistasis
**Psyn1:1**	F_v/F_m	1	3.0	1.4	6.2	6.45	2.78	-0.0078	0.0015	
**Psyn1:2**	NPQ_{fast}	1	13.5	12.6	14.5	7.13	2.41	0.0339	0.0060	
	NPQ_{slow}	1	13.5	12.6	14.1	18.09	8.52	0.0274	0.0030	
**Psyn1:3**	NPQ_{fast}	1	26.5	22.7	33.8	2.79	0.92	0.0224	0.0062	
**Psyn1:4**	NPQ_{slow}	1	61.1	30.9	83.8	2.76	1.21	0.0110	0.0030	
	NPQ_{fast}	1	61.1	58.8	62.5	5.11	1.71	0.0343	0.0069	
**Psyn1:5**	NPQ_{fast}	1	68.4	67.6	70.4	14.32	5.00	-0.0396	0.0068	w/ Psyn5:2
**Psyn1:6**	F_v/F_m	1	79.6	77.2	83.8	5.82	2.50	0.0072	0.0015	
**Psyn2:1**	NPQ_{fast}	2	25.6	12.8	32.6	5.36	1.80	-0.0332	0.0065	
**Psyn2:2**	F_v/F_m	2	35.6	34.3	37.4	6.62	2.86	0.0084	0.0016	
	NPQ_{fast}	2	35.6	35.6	37.4	8.44	2.87	0.0419	0.0066	
**Psyn2:3**	F_v/F_m	2	58.0	44.3	60.9	3.11	1.32	-0.0054	0.0016	
**Psyn3:1**	NPQ_{slow}	3	4.5	1.0	6.3	12.68	5.82	0.0220	0.0031	
	NPQ_{fast}	3	6.3	4.5	7.3	8.06	2.73	0.0329	0.0055	
**Psyn3:2**	F_v/F_m	3	18.0	18.0	19.7	17.28	7.85	0.0142	0.0016	
	NPQ_{fast}	3	24.4	19.7	28.0	4.64	1.55	0.0282	0.0062	
**Psyn3:3**	NPQ_{fast}	3	45.9	45.9	49.1	34.02	13.07	0.0801	0.0058	
	F_v/F_m	3	47.5	45.9	49.1	16.62	7.52	0.0139	0.0016	
	NPQ_{slow}	3	47.5	45.9	49.1	31.20	15.66	0.0429	0.0035	
**Psyn3:4**	NPQ_{slow}	3	70.5	63.6	70.5	5.50	2.44	-0.0172	0.0034	
	NPQ_{fast}	3	70.5	68.4	70.5	5.86	1.97	-0.0278	0.0052	
**Psyn4:1**	NPQ_{slow}	4	42.3	33.4	52.4	7.45	3.34	0.0178	0.0031	
**Psyn5:1**	NPQ_{fast}	5	5.7	3.9	6.7	9.55	3.26	0.0375	0.0057	
**Psyn5:2**	F_v/F_m	5	20.4	11.4	27.1	5.88	2.53	-0.0074	0.0015	
	NPQ_{slow}	5	20.4	15.9	22.0	5.38	2.39	-0.0155	0.0032	
	NPQ_{fast}	5	20.4	20.4	20.4	17.53	6.22	-0.0454	0.0059	w/ Psyn1:5
**Psyn5:3**	NPQ_{fast}	5	50.5	50.5	51.0	39.22	15.46	0.0722	0.0050	
**Psyn5:4**	NPQ_{slow}	5	56.6	56.6	58.6	29.37	14.61	0.0366	0.0030	
**Psyn5:5**	F_v/F_m	5	66.1	64.6	66.5	10.50	4.62	0.0141	0.0021	
**Psyn5:6**	F_v/F_m	5	74.5	70.1	78.2	2.98	1.27	0.0076	0.0021	
